# DANCE AND EMOTION: THE IMPACT OF OLDER PERFORMERS ON AUDIENCE ENGAGEMENT

**DOI:** 10.1093/geroni/igae098.0202

**Published:** 2024-12-31

**Authors:** Atsuko Miyazaki

**Affiliations:** The University of Tokyo, Meguro-ku, Tokyo, Japan

## Abstract

This study explores the emotional connections between dancers of advanced age and their audience, investigating the role of dance in promoting happiness and social engagement among older adults. The Federation International of Dancers Association Japan (FIDA JAPAN), a general incorporated association supporting dance activities for those aged 65 and above, has been actively launching hip-hop dance teams for older adults across Japan since 2021. In September 2023, the FIDA GOLD CUP 2023 dance competition was held, featuring 13 teams from various regions in Japan. Among the dancers who participated in the competition and responded to the post-performance survey (n=148, mean age=70.99 years, SD=5.46), 62.8% started dancing at the age of 65 or older. Audience members (n=81, mean age=51.69 years, SD=15.85) were also surveyed. Both surveys assessed various aspects of emotional experiences using a 5-point scale. Analysis revealed that dancers reported significantly higher levels of emotional resonance (M=5.24, SD=0.82) compared to the audience (M=4.68, SD=0.62), t(227)=5.58, p< 0.001, Cohen’s d=0.77, indicating a large effect size. Additionally, a statistically significant positive correlation was found between dancers’ feelings of exhilaration on stage and the audience’s applause (ρ=0.31, p< 0.01), suggesting that the emotional experiences of dancers and their audience are interconnected. The results highlight the emotional interplay between dancers of advanced age and their audience, emphasizing the potential for dance to foster a shared emotional experience. The findings contribute to understanding how dance can enhance emotional well-being and social engagement, particularly for those who take up dancing in later life.

